# Kidney Organoids as a Novel Platform to Evaluate Lipopolysaccharide-Induced Oxidative Stress and Apoptosis in Acute Kidney Injury

**DOI:** 10.3389/fmed.2021.766073

**Published:** 2021-11-29

**Authors:** Weitao Zhang, Ruochen Qi, Tingting Li, Xuepeng Zhang, Yi Shi, Ming Xu, Tongyu Zhu

**Affiliations:** ^1^Department of Urology, Zhongshan Hospital, Fudan University, Shanghai, China; ^2^Shanghai Key Laboratory of Organ Transplantation, Shanghai, China; ^3^Institute of Clinical Science, Zhongshan Hospital, Fudan University, Shanghai, China; ^4^Department of Critical Care Medicine, Zhongshan Hospital, Fudan University, Shanghai, China

**Keywords:** kidney organoids, human pluripotent stem cells, sepsis, acute kidney injury, oxidative stress, apoptosis

## Abstract

Sepsis-associated acute kidney injury (SA-AKI) is a life-threatening syndrome. Lipopolysaccharide (LPS) is a widely used inducer for modeling SA-AKI both *in vivo* and *in vitro*. However, due to the innate complexity of the kidney architecture, the mechanisms underlying the pathogenesis of SA-AKI, as well as those involved in LPS-induced kidney injury remain to be clarified. Kidney organoids derived from human pluripotent stem cells (hPSCs) act as a model of multiple types of kidney cells *in vitro* and eliminate potential confounders *in vivo*. In the current study, we established LPS-induced kidney injury models both *in vivo* and in human kidney organoids. Kidney function, pathological changes, and markers of oxidative stress were evaluated with/without the presence of methylprednisolone (MP) treatment both *in vivo* and *in vitro*. The extent of LPS-induced oxidative stress and apoptosis in kidney organoids was further investigated *in vitro*. LPS-induced acute kidney injury in mice, together with pathological changes and increased oxidative stress, as well as enhanced apoptosis in kidney cells were evaluated. These phenomena were ameliorated by MP treatment. Experiments in kidney organoids showed that the LPS-induced apoptotic effects occurred mainly in podocytes and proximal tubular cells. Our experiments demonstrated the efficacy of using kidney organoids as a solid platform to study LPS-induced kidney injury. LPS induced oxidative stress as well as apoptosis in kidney cells independently of changes in perfusion or immune cell infiltration. MP treatment partially alleviated LPS-induced injury by reducing kidney cell oxidative stress and apoptosis.

## Introduction

Sepsis is characterized by a set of clinical syndromes caused by the dysregulated host response to infection and is manifested by organ dysfunction, according to the definition of the Third International Consensus (Sepsis-3) (1, 2). Sepsis represents a leading cause of death in critically ill patients. The kidney is one of the most vulnerable organs affected during the progression of sepsis. Sepsis-associated acute kidney injury (SA-AKI), like acute kidney injury (AKI) due to other causes, is characterized by an increase in serum creatinine levels and/or by a decrease in urine volume (2–4). Owing to shared pathophysiological mechanisms, sepsis and AKI are inseparable and should be considered as integral entities in the clinical setting. In fact, sepsis and AKI exert increased host susceptibility to either condition. With sepsis being the leading cause of the development of AKI, AKI, in turn, also enhances the risk of progression of sepsis (5, 6). Currently, the underlying pathophysiological mechanisms of SA-AKI are largely unknown, and effective therapy for treatment is still absent (7). Thus, it is of great importance to explore the innate mechanism of SA-AKI and to provide evidence for treatment in the clinical setting.

Lipopolysaccharide (LPS), a constituent of the cell wall of Gram-negative bacteria, has been widely used to induce SA-AKI in animal models. However, due to the complex pathogenesis of SA-AKI, including hemodynamic alterations, microvascular dysfunction, the generation of reactive oxygen species (ROS), inflammation, and metabolic reprogramming, previous research has yielded different or even controversial results regarding the mechanism of development of SA-AKI (8, 9). For example, Lee et al. found that tubular epithelial cell apoptosis plays a central role in the development of SA-AKI (10); however, another study did not find any differences in the apoptosis rate in an SA-AKI sheep model compared with the control counterparts (11). These controversies could be partially attributed to the complicated hemodynamic changes and the intricate immune microenvironment within the kidney (7). Other studies chose to switch to *in vitro* experiments to eliminate these potential confounders. Most studies have been carried out using proximal tubular epithelial cells to study the toxicity of LPS (12). However, the kidney is made up of a variety of cell types, including but not limited to podocytes, proximal tubular cells, loops of Henle cells, distal tubular cells, and collecting duct cells. Therefore, the use of a single or a few different cell lines does not accurately reflect the comprehensive activity of LPS; therefore, additional caution should be taken when interpreting these findings in clinical settings.

Kidney organoids derived from human pluripotent stem cells (hPSC) contain multiple cell types and could effectively model the complexity of the kidney structure (13–16). In addition, kidney organoids are easier to manipulate than animal models and could exclude the confounding effects of hemodynamic alternation as well as immune cell infiltration (17, 18). Thus, maintaining the complexity of different cell types and human genome characteristics, while processing in culture dishes in a high-throughput manner allows kidney organoids to be an ideal model for studying the physiological and pathological changes of the kidney.

In this study, we established an SA-AKI mouse model induced by LPS and applied kidney organoids to study the direct toxicity of LPS, especially the mechanisms involving oxidative stress and apoptosis in kidney cells *in vitro*. In these models, we also used methylprednisolone (MP), a widely used anti-inflammatory drug for the treatment of sepsis, and investigated the potential therapeutic mechanism of MP in SA-AKI.

## Materials and Methods

### Establishment of an LPS-Induced Mice Kidney Injury Model

Male BALB/c mice, aged 6–8 weeks, were acquired from the Shanghai JSJ Lab Animal, Co. Ltd. (China) and were housed in a specific-pathogen-free (SPF) grade animal room with free access to food and water. The mice were fasted for 12 h before treatment and then randomized to the control group, LPS treatment group (with different treatment times), and LPS + MP treatment group (*n* = 6 for each group). LPS was dissolved in 0.9% saline and administered intraperitoneally at a dose of 10 mg/kg (19). MP was first reconstituted in a 0.9% benzyl alcohol solution (provided in the original bottle) for storage. The MP solution was further dissolved in 0.9% saline and administered at a dose of 5 mg/kg intraperitoneally after LPS treatment. The same amount of saline was given to the control group. The mice were euthanized after the corresponding treatment time. All protocols were carried out according to the Guidelines for the Care and Use of Laboratory Animals of the Fudan University Laboratory Animal Ethical Commission and approved by the Animal Ethical Committee of Zhongshan Hospital, Fudan University (No. 2018-046).

### Differentiation of Kidney Organoid and Treatment

The hPSC cell line H9 was acquired from Shanghai Zhong Qiao Xin Zhou Biotechnology Co. Ltd. (China) and cultured in 6-well Matrigel Matrix coated plates with a mTeSR1 medium. The differentiation of kidney organs was carried out according to a previously published protocol (20). Briefly, the hPSC cells were harvested, resuspended with 10 μM Y27632, and seeded in a 24-well Matrigel matrix-coated plate. Treatment with 8 μM CHIR99021 was added to the culture system for 4 days (to Day 4), followed by 3 days of 10 mg/mL Activin A induction (to Day 7), and then 2 days of 10 ng/mL FGF9 induction (to Day 9). The cells were reseeded into an ultralow attachment 96-well plate on day 9 with continued induction with FGF9 to day 14. The culture medium was then switched to Advanced RPMI-1640 until day 21. The kidney organoids should be matured on day 21 after seeding.

After seeding, the kidney organoids were randomized to the control group, LPS treatment group (with different concentrations of LPS), and LPS+MP treatment group. LPS and MP were reconstituted with phosphate-buffered saline (PBS) and then dissolved in an RPMI-1640 medium. The dose of LPS ranged from 100 ng/mL to 10 μg/mL and the dosage of MP was 1 μg/mL, and the treatment time was 24 h. A total of 242 kidney organoids were used in the *in vitro* experiments. Specifically, 30 organoids were used to identify markers of renal components and the presence of Toll-like receptor 4 (TLR4). For the immunofluorescence experiment, 12 organoids were randomly assigned to the three groups (*n* = 4 for each group). For the experiments evaluating the malondialdehyde (MDA) enzyme activity and superoxide dismutase (SOD) activity, four kidney organoids were placed in a single sample for protein extraction, and nine samples were allocated to the three groups for each parameter (*n* = 3 for each group). For real-time qPCR, four kidney organoids were placed in one single sample for RNA extraction, and 12 samples were divided into three groups for the subsequent experiments (*n* = 4 for each group).

### Hematoxylin and Eosin Staining, Terminal Deoxynucleotidyl Transferase DUTP Nick End Labeling Assay, Immunohistochemistry, and Immunofluorescence Staining

The mice kidney tissue was fixed with 4% paraformaldehyde and then embedded in paraffin. Sections (3 μm) were obtained from the embedded tissues. H&E staining was performed according to standard procedures. The TUNEL assay kit was purchased from Roche (Basel, Switzerland) and the assay was performed according to the protocol of the manufacturer. For the immunohistochemical staining, anti-F4/80 (Cell Signaling Technology, Danvers, MA, USA) and anti-myeloperoxidase (MPO) antibodies (Abcam, Cambridge, UK) were added to the kidney sections at four overnights. On the second day, the sections were washed with tris-buffered saline (TBST) three times before incubation with HRP-conjugated IgG H&L at 37°C for 45 min. 3-diaminobenzidine HCl (DAB) was used for staining for 5 min. The immunofluorescence staining of the kidney organoids was performed according to a previously published protocol (20). A positive expression in immunohistochemistry and immunofluorescence staining was determined and analyzed using ImageJ (version 1.52a, National Institutes of Health, Bethesda, MD, USA).

### Evaluation of Oxidative Stress

The kidney tissue and kidney organoid samples were harvested 24 h after the corresponding treatment. The MDA and SOD enzyme activity were determined with commercial kits according to the instructions of the manufacturer (both purchased from Beyotime Biotechnology, Shanghai, China).

For the high-content imaging analysis, fresh kidney organoids were incubated with dihydroethidium (DHE) (Beyotime Biotechnology, Shanghai, China) and CellRox Green (Invitrogen, Waltham, MA, USA) for 30 min. The kidney organoids were subsequently washed with PBS twice and transferred to a live-cell imaging solution for observation using the Operetta^®^ High Content Screening System (Perkin Elmer, Waltham, MA, USA).

### RNA Extraction and Real-Time Quantitative Polymerase Chain Reaction

The total RNA from the kidney organoids was extracted using a Trizol (Sigma-Aldrich, Tokyo, Japan) and Zymo-Spin column (Zymo Research, Irvine, CA, USA). qPCR was carried out using the iScript cDNA synthesizer kit and the SYBR green Master Mix (both from Bio-rad, Hercules, CA, USA) according to the instructions of the manufacturer. The primers used are listed in Table 1.

**Table 1:** Primers used for quantitative real-time PCR analysis of human kidney organoids.

**GENE**		**Primer sequence**
*NPHS1*	Forward	CTGCCTGAAAACCTGACGGT
	Reverse	GACCTGGCACTCATACTCCG
*NPHS2*	Forward	ACCAAATCCTCCGGCTTAGG
	Reverse	CAACCTTTACGCAGAACCAGA
*PODXL*	Forward	TCCCAGAATGCAACCCAGAC
	Reverse	GGTGAGTCACTGGATACACCAA
*HNF4A*	Forward	CGAAGGTCAAGCTATGAGGACA
	Reverse	ATCTGCGATGCTGGCAATCT
*GATA3*	Forward	GCCCCTCATTAAGCCCAAG
	Reverse	TTGTGGTGGTCTGACAGTTCG
*CDH1*	Forward	CGAGAGCTACACGTTCACGG
	Reverse	GGGTGTCGAGGGAAAAATAGG
*HAVCR1*	Forward	TGGCAGATTCTGTAGCTGGTT
	Reverse	AGAGAACATGAGCCTCTATTCCA
*GAPDH*	Forward	CCCATCACCATCTTCCAGGAG
	Reverse	CTTCTCCATGGTGGTGAAGACG

### Reagents

The following reagents were used for this study: mTSER1 medium, accutase, and Y-27632 (Stem Cell Technology, Seattle, WA, USA); Advanced RPMI 1640 medium (Gibco, Waltham, MA, USA); CHIR 99021 (Cayman, Ann Arbor, MI, USA); Activin A and FGF9 (Peprotech, Cranbury, NJ, USA); LPS (Sigma-Aldrich, Tokyo, Japan); Methylprednisolone (Pfizer, New York, NY, USA), Anti-GATA3 antibody and anti-cleaved Caspase-3 antibody (Cell Signaling Technology, Danvers, MA, USA); anti-HNF4α antibody, anti-synaptopodin antibody (Santa Cruz Biotechnology, Santa Cruz, CA, USA); anti-PAX2 antibody and anti-podocalyxin antibody (R&D Systems, Minneapolis, MN, USA), LTL fluorescence dye (Vector Laboratories, Burlingame, CA, USA); anti-E-cadherin antibody (BD Pharmingen, San Jose, CA, USA); and anti-TLR4 antibody and anti-Nrf2 antibody (Affinity Biosciences, Cincinnati, OH, USA).

### Statistical Analysis

The data were presented as mean ± SD. Statistical analysis was performed using GraphPad Prism 8 (GraphPad Software Inc., San Diego, CA, USA). The Student's *t*-test was performed for comparison between two groups. One-way ANOVA was used for comparisons among multiple groups. Statistical significance was established as *P* < 0.05.

## Results

### MP Treatment Improved LPS-Induced AKI *in vivo*

**Figure 1:**
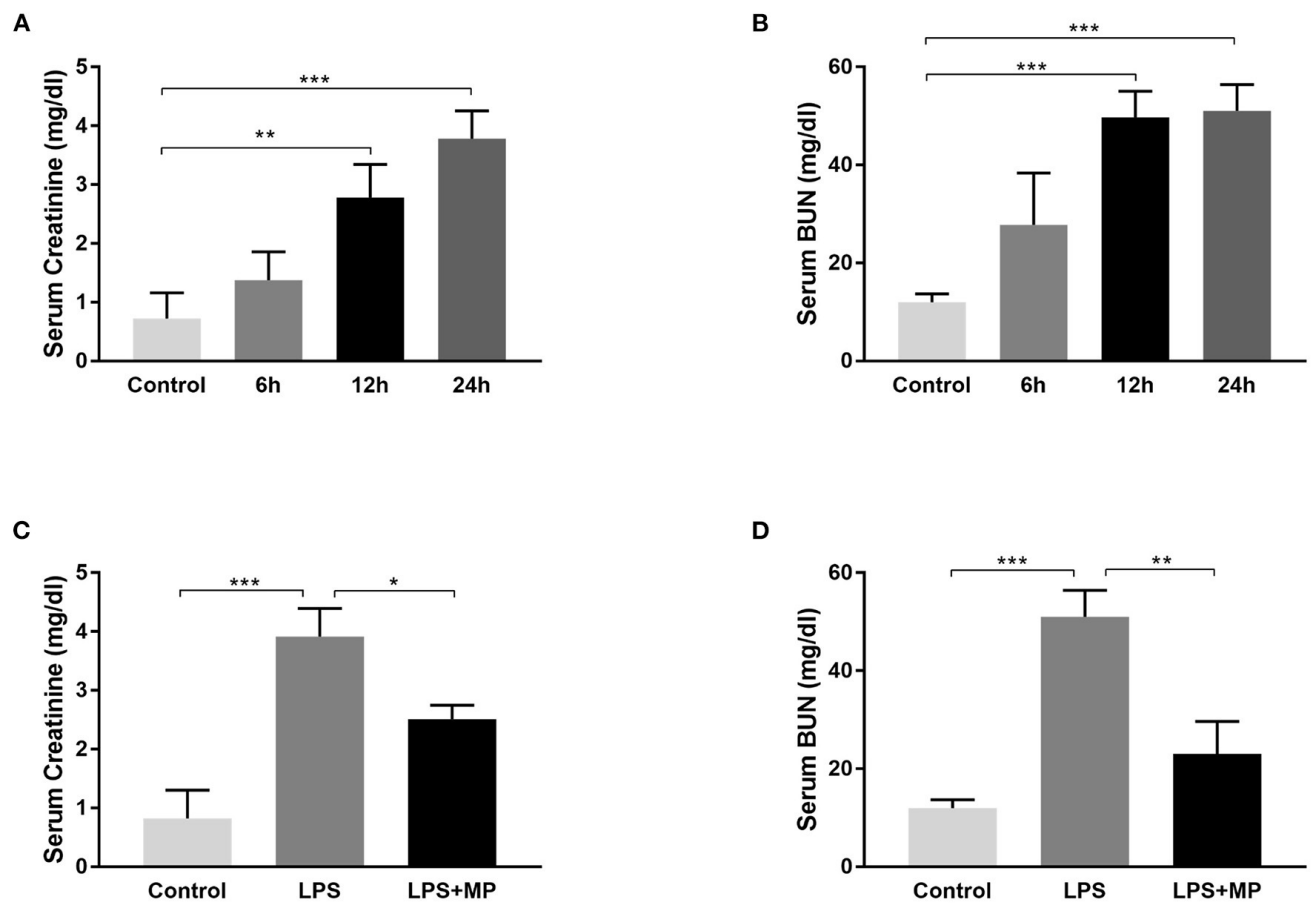
Lipopolysaccharide (LPS) induces acute kidney injury (AKI) in mice and was ameliorated by methylprednisolone (MP) treatment. **(A)** Serum creatinine levels and **(B)** serum blood urine nitrogen (BUN) levels of mice treated with LPS for different periods. **(C)** Serum creatinine levels and **(D)** serum BUN levels of mice treated with LPS and MP. ^*^P < 0.05, ^**^P < 0.01, ^***^P < 0.001.

Lipopolysaccharide is widely used for SA-AKI induction in mice (12). We verified its efficacy by injecting 10 mg/kg of LPS (dissolved in 0.9% saline) into mice, intraperitoneally. The evaluation of the blood samples showed that the serum creatinine level and blood urine nitrogen (BUN) levels continued to increase after LPS injection and reached a peak at 24 h after induction (Figures 1A,B), indicating deteriorated kidney function. Therefore, we chose 24 h as the LPS induction time in the subsequent *in vivo* experiments.

Methylprednisolone is a glucocorticoid that is frequently used to treat sepsis in the clinical setting (21), due to its powerful anti-inflammatory effect. We also evaluated its efficacy in LPS treated mice. We observed a decrease in serum creatinine and BUN level after MP treatment (Figures 1C,D), indicating its potential therapeutic effects on SA-AKI.

### MP Partially Reversed Histological Changes and Oxidative Stress Caused by LPS Induction

**Figure 2:**
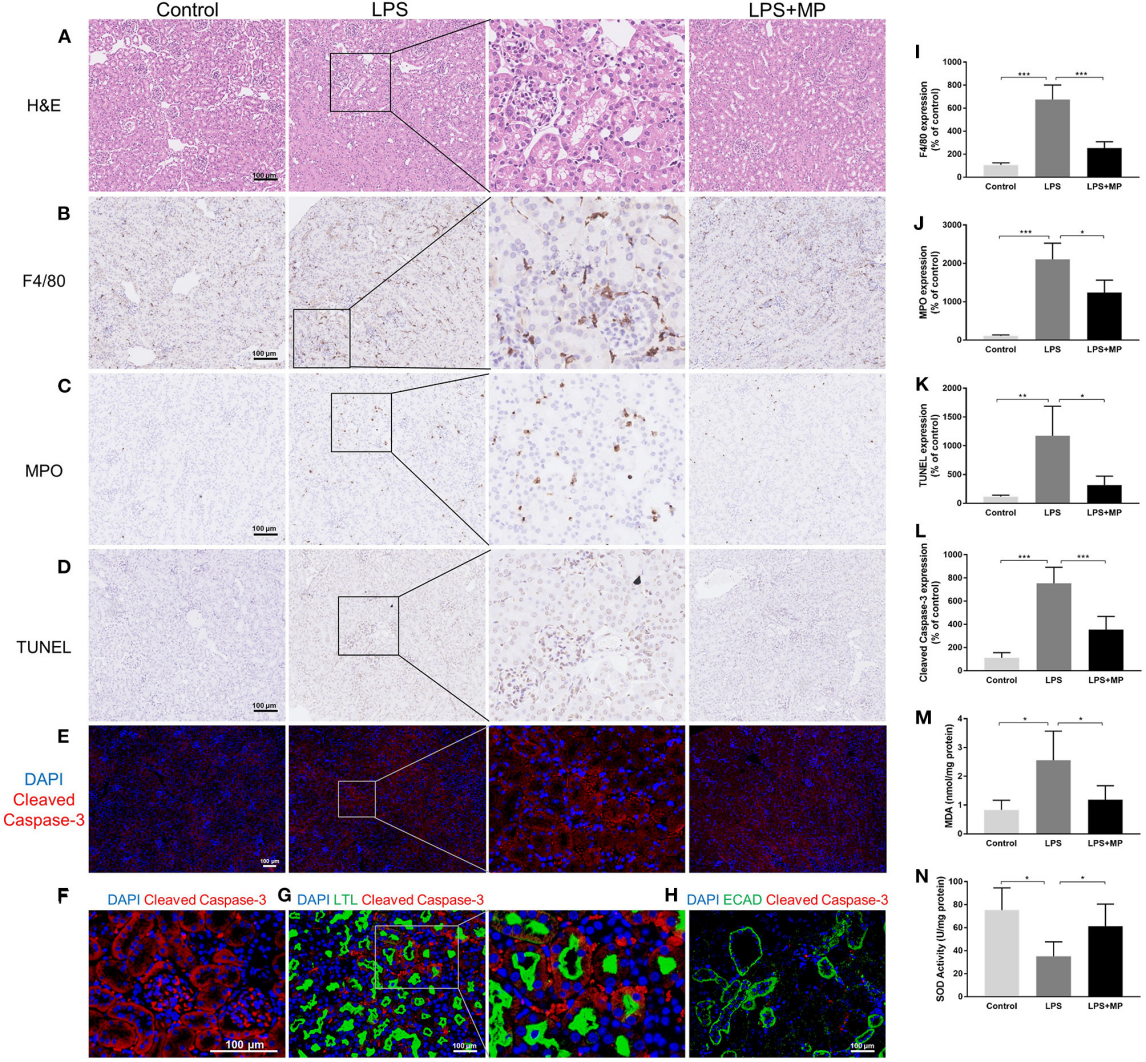
LPS induces pathological changes as well as oxidative stress in the kidney and was ameliorated by MP treatment. **(A)** Representative images of hematoxylin and eosin (H&E) staining of kidney sections from the control, LPS, and LPS + MP treatment group. **(B)** Representative images of immunohistochemistry staining of the macrophage marker, F4/80 in kidney sections. **(C)** Representative images of immunohistochemistry staining of the neutrophil marker, myeloperoxidase (MPO) in kidney sections. **(D)** Representative images of the terminal deoxynucleotidyl transferase dUTP nick end labeling (TUNEL) assay and **(E)** cleaved Caspase-3 (Red) in kidney sections. **(F)** Magnification of cleaved Caspase-3 (Red) expression in the glomerular area of the LPS group. **(G)** Expression pattern of the proximal tubular marker Lotus Tetragonolobus Lectin (LTL) (Green) as well as cleaved Caspase-3 (Red) in the LPS group. **(H)** Expression pattern of the distal tubular marker ECAD (Green) as well as cleaved Caspase-3 (Red) in the LPS group. The 6-diamidino-2-phenylindole (DAPI) is stained with blue fluorescence. The corresponding semi-quantitative analysis of **(I)** F4/80, **(J)** MPO, **(K)** TUNEL, and **(L)** cleaved Caspase-3. **(M)** MDA concentration of mice kidney tissues. **(N)** SOD activity of mice kidney tissues. Scar bar = 100 μm. ^*^P < 0.05, ^**^P < 0.01, ^***^P < 0.001.

The H&E staining revealed cellular swelling in the LPS treatment group; however, the glomerular and tubular structure generally remained intact. The MP treatment partially ameliorated this phenomenon (Figure 2A).

Previous studies demonstrated that inflammation played an important role in the development of SA-AKI (2). We performed immunohistochemical staining to detect F4/80, a macrophage marker, and MPO, a neutrophil marker, in the kidney sections. We found that LPS exacerbated the macrophage and neutrophil infiltration, while the MP treatment decreased this, further confirming the anti-inflammatory effects of MP (Figures 2B,C,I,J).

Intact parenchymal cells are essential for normal kidney structure and function (22). We evaluated the kidney cell apoptosis using the TUNEL assay and detected the expression of cleaved Caspase-3 in kidney sections. We found that apoptosis increased significantly after LPS induction, in both glomerular cells and tubular cells, and was partially alleviated by MP treatment (Figures 2D,E,K,L). We further evaluated the expression of cleaved Caspase-3 in the glomerular area and colocalized the expression of cleaved Caspase-3 with Lotus Tetragonolobus Lectin (LTL), a murine proximal tubular marker, and E-cadherin (ECAD), a distal tubular marker, respectively. The results indicated that LPS-induced apoptosis mainly affected the glomerular areas and the proximal tubules in mice, but not distal tubules (Figures 2F–H).

The overproduction of ROS contributes to increased oxidative stress, which is also a well-known mechanism of SA-AKI (23). We detected the expression of MDA, a parameter of oxidative stress, and SOD, an endogenous antioxidant in kidney tissue. We found an elevation of MDA and a decrease in SOD activity caused by LPS, while MP reversed these trends (Figures 2M,N).

### hPSC-Derived Kidney Organoids Contained Different Renal Components and Expressed TLR4

In our previous experiments, we observed kidney injury and apoptosis after LPS treatment. However, it was difficult to elucidate whether kidney injury was induced by the direct effect of LPS toxicity since the kidney was composed of not only parenchymal cells but also included an influx of inflammatory cells. hPSC-derived kidney organoids allowed us to model the various types of kidney cells *in vitro*, while, excluding the influence of the influx of circulating inflammatory cells.

**Figure 3:**
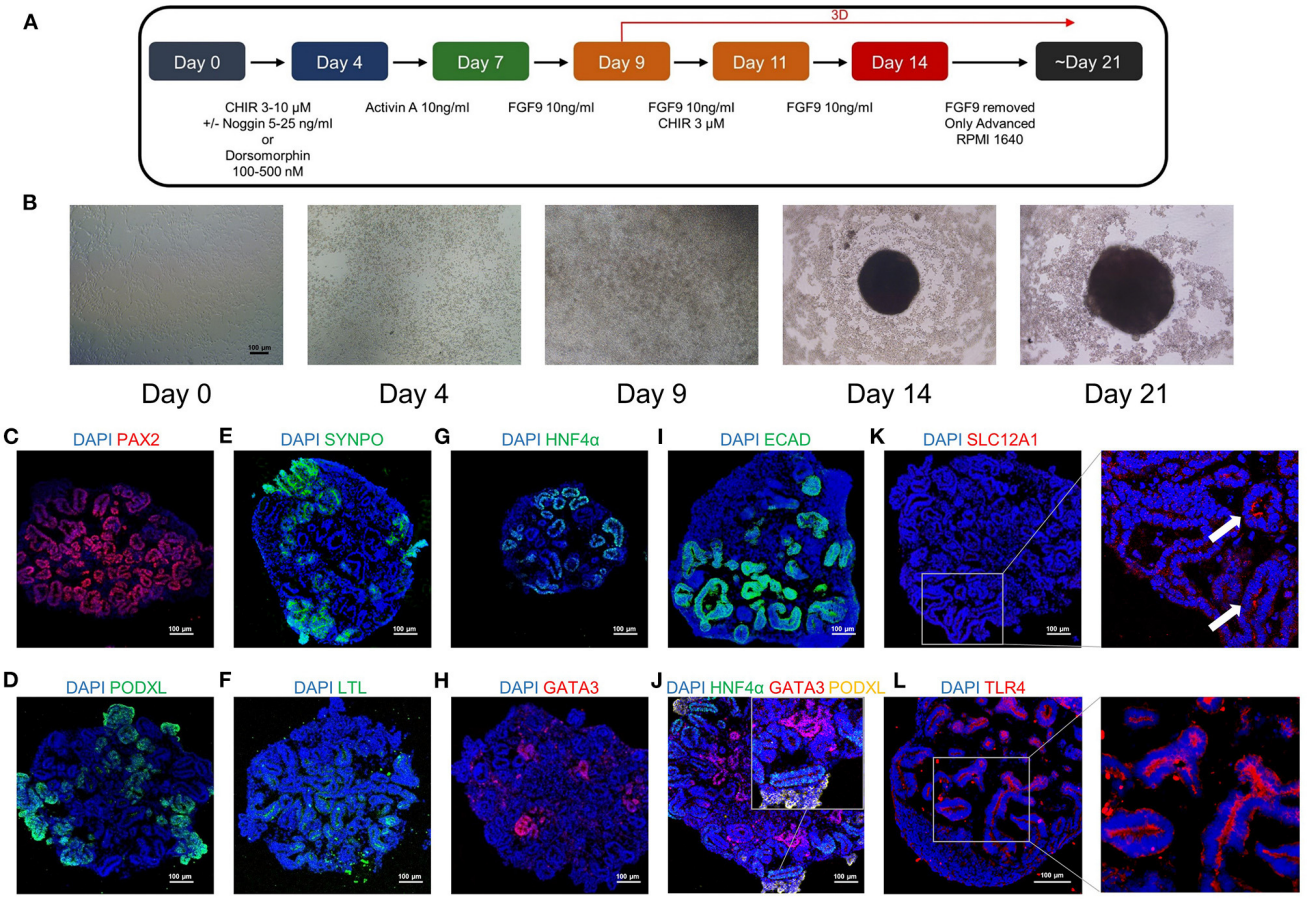
The human pluripotent stem cells (hPSC)-derived kidney organoids contain different renal structures and expressed TLR4. **(A)** A schematic overview of the kidney organoids differentiation protocol. **(B)** Morphological changes of the kidney organoids differentiation under bright field observation. Kidney organoids in this study expressed the intermediate mesoderm marker **(C)** PAX2 (Red), the podocyte markers **(D)** podocalyxin (PODXL) (Green) and **(E)** synaptopodin (SYNPO) (Green), the proximal tubule markers **(F)** LTL (Green) and **(G)** HNF4α (Green), the distal tubule as well as collecting duct markers **(H)** GATA3 (Red), and **(I)** E-cadherin (ECAD) (Green). **(J)** The association between HNF4α (Green), GATA3 (Red) and PODXL (Yellow) in the kidney organoids. The expression pattern of the loop of Henle marker **(K)** SLC12A1 (Red) and the LPS receptor **(L)** TLR4 (Red) in the kidney organoids. DAPI is stained with blue fluorescence. Scar bar = 100 μm.

We induced kidney organoids with H9 cells based on a well-published protocol (Figure 3A). Under microscopic bright field observation, the kidney organoid was differentiated from two-dimensional cells to three-dimensional spheroids in the 96-well ultra-low attachment plate, and then grew to a larger size at day 21, the endpoint of differentiation (Figure 3B). Further immunofluorescence staining on kidney organoids revealed the expression of the intermediate mesoderm marker PAX2 (Figure 3C), the podocyte markers podocalyxin (PODXL) and synaptopodin (SYNPO) (Figures 3D,E), the proximal tubule markers LTL and HNF4α (Figures 3F,G), and distal tubule, as well as collecting duct markers GATA3 and E-cadherin (ECAD) (Figures 3H,I). An association between HNF4α, GATA3, and PODXL was also identified (Figure 3J). Furthermore, the expression of SLC12A1 was found in the kidney organoids, indicating the presence of the cells of the loop of Henle (Figure 3K). TLR4 is the fundamental receptor for LPS and mediates damage to the kidney when activated by LPS (24). Furthermore, we confirmed that TLR4 was widely expressed in kidney organoids (Figure 3L). These results indicated that kidney organoids contained various renal parenchymal components and expressed TLR4, which allowed kidney organoids to be qualified for the investigation of LPS-induced kidney injury.

### LPS Treatment Did Not Cause Obvious Morphological Changes, but Induced Apoptosis in Kidney Organoids

**Figure 4:**
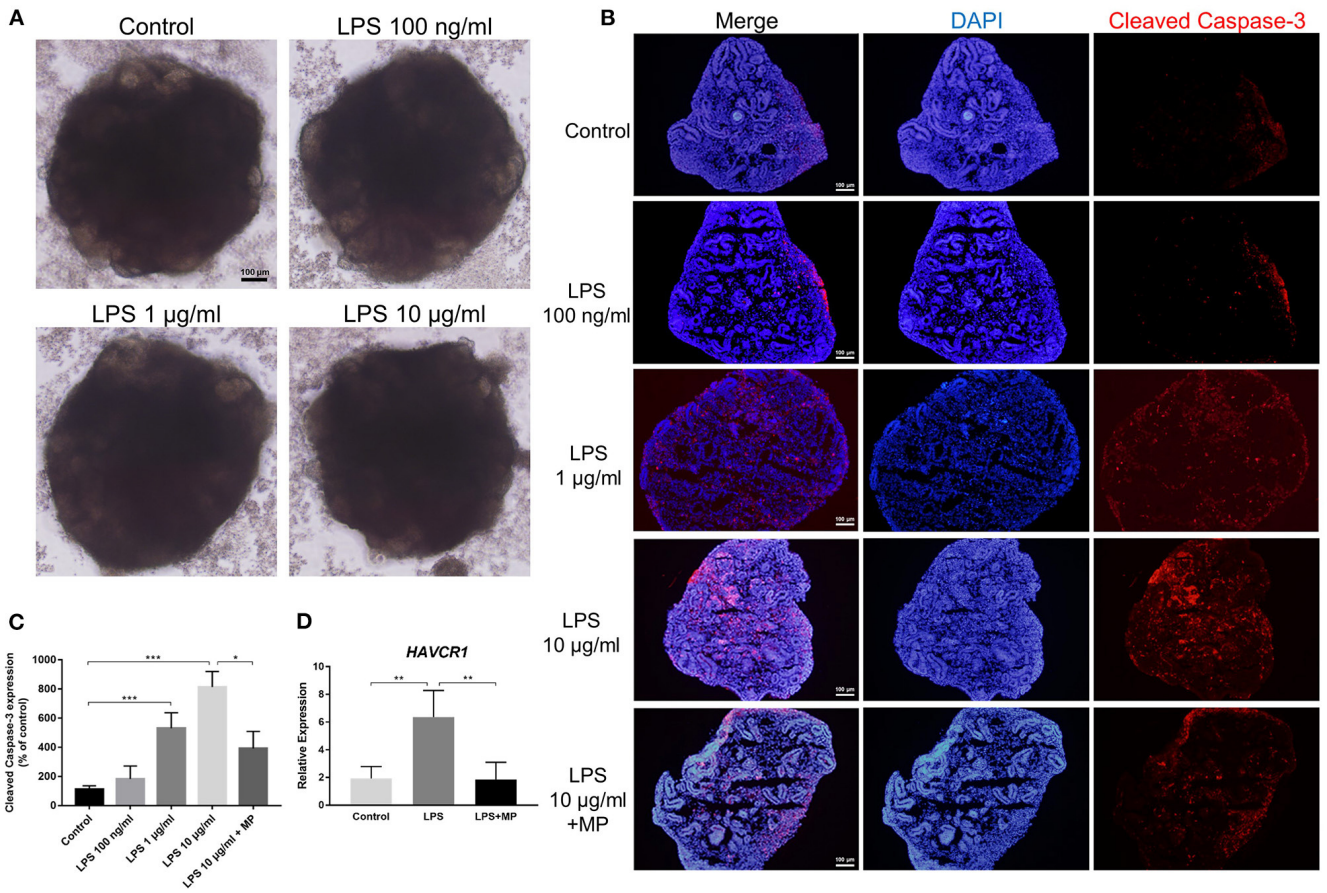
LPS does not alter the structure but induces apoptosis in kidney organoids. **(A)** Different concentrations of LPS caused no morphological changes in kidney organoids. **(B)** LPS induced apoptosis in kidney organoids in a dose-dependent manner and was alleviated by MP treatment. Kidney organoids were stained with cleaved Caspase-3 (Red) and DAPI (Blue). **(C)** Corresponding semi-quantitative analysis of cleaved Caspase-3. **(D)** Quantitative PCR analysis of the AKI marker HAVCR1. Scar bar = 100 μm. ^*^P < 0.05, ^**^P < 0.01, ^***^P < 0.001.

We treated mature kidney organoids with different concentrations of LPS, ranging from 100 ng/mL to 10 μg/mL (reconstituted in PBS and dissolved in the corresponding medium) for 24 h. However, we did not observe obvious morphological changes in the LPS-treated groups under brightfield microscopy, spherical structures, as well as the microscopic tubular structures, remained intact after LPS treatment (Figure 4A).

In the *in vivo* study, we found that LPS treatment caused apoptosis in kidney cells. Next, we investigated whether this apoptotic effect was caused directly by LPS toxicity or by other secondary damage. Immunofluorescence showed that the expression of cleaved Caspase-3, the marker of activated apoptosis, gradually increased with the increasing concentrations of LPS treatment. Since 10 μg/mL LPS treatment caused the most significant changes, we chose this dosage in the following *in vitro* experiments. Treatment with MP significantly reduced the expression of cleaved Caspase-3 in kidney organoids (Figures 4B,C). This showed that LPS could exert a direct effect on kidney cell apoptosis. In other words, the apoptosis of kidney cells, at least partially, is derived from the direct effect of LPS. Furthermore, to verify the existence of kidney injury, we performed qPCR with a typical AKI marker HAVCR1 (encoding KIM-1) and found that LPS caused a notable increase in HAVCR1 expression in kidney organoids (Figure 4D). Furthermore, the MP treatment counteracted the effect of LPS.

### LPS Increased Oxidative Stress in Kidney Organoids

**Figure 5:**
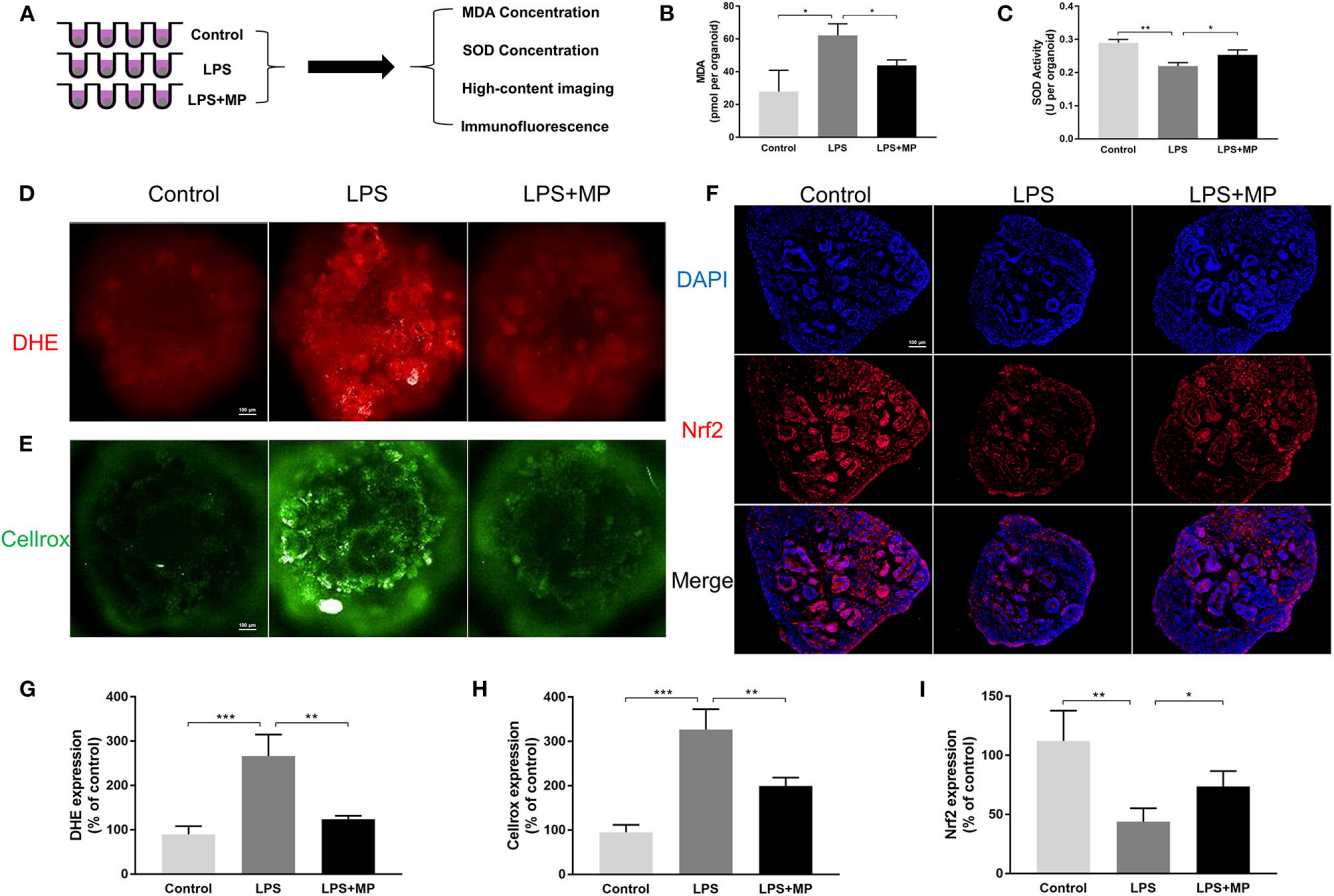
LPS enhances oxidative stress in kidney organoids and is alleviated by MP. **(A)** Schematic representation of the study design of oxidative stress evaluation in kidney organoids. **(B)** malondialdehyde (MDA) concentration of kidney organoids treated with LPS and MP. **(C)** superoxide dismutase (SOD) activity of kidney organoids treated with LPS and MP. **(D)** dihydroethidium (DHE) and **(E)** Cellrox staining of live kidney organoids under high-content imaging analysis. **(F)** Expression pattern of Nrf2 (Red) in the control, LPS, and LPS + MP treatment group. The corresponding semi-quantitative analysis of **(G)** DHE **(H)** Cellrox and **(I)** Nrf2. Scar bar = 100 μm. ^*^P < 0.05, ^**^P < 0.01, ^***^P < 0.001.

Excess oxidative stress has been reported to induce both an immune response and intrinsic pathological injury to organs (25). In addition, oxidative stress can be a direct trigger for apoptosis in the kidney (26). We conducted a series of experiments to detect oxidative stress in kidney organoids (Figure 5A). The expression pattern of MDA and SOD in kidney organoids was similar to that *in vivo* (Figures 5B,C). Furthermore, high-content imaging analysis was used to determine the level of oxidative stress in live kidney organoids. The expression of DHE and Cellrox increased markedly after LPS treatment, while they were again alleviated by MP (Figures 5D,E,G,H).

Nuclear factor erythroid-2 (NF-E2) related factor 2 (Nrf2) is widely recognized as a vital regulator of redox homeostasis (27). Numerous studies have confirmed the beneficial role of Nrf2 in kidney damage. Thus, we evaluated Nrf2 in kidney organoids. The Nrf2 expression was markedly reduced by LPS, while MP could counteract this effect (Figures 5F,I), which also indicated that MP reduced oxidative stress independently of inflammatory cell influx.

### LPS Induced Apoptosis Mainly in Podocytes and Proximal Tubular Cells

**Figure 6:**
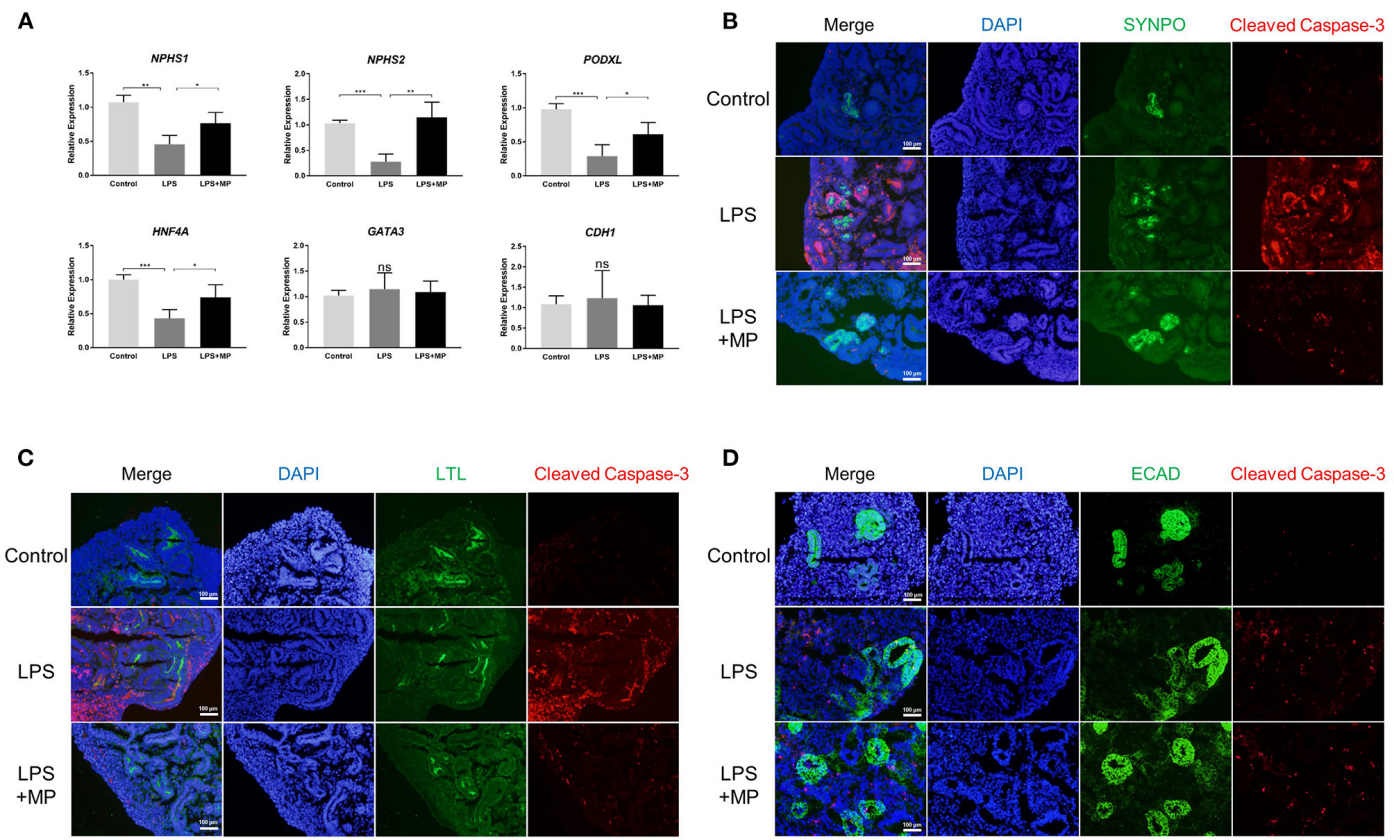
LPS induces apoptosis mainly in podocytes and proximal tubular cells, but not in distal tubular cells. **(A)** Quantitative PCR analysis of the podocytes markers (NPHS1, NPHS2, PODXL), proximal tubular marker (HNF4A), and distal tubular markers (GATA3, CDH1). ns, non-significant, ^*^P < 0.05, ^**^P < 0.01, ^***^P < 0.001. **(B)** Expression pattern of the podocyte marker SYNPO (Green) as well as cleaved Caspase-3 (Red) in the control, LPS, and LPS + MP treatment group. **(C)** Expression pattern of the proximal tubular marker LTL (Green) as well as cleaved Caspase-3 (Red). **(D)** Expression pattern of the distal tubular marker ECAD (Green) as well as cleaved Caspase-3 (Red). DAPI is stained with blue fluorescence. Scar bar = 100 μm.

To further elucidate the role of apoptosis in kidney organoids, we detected the expression of different cell markers using qPCR in the organoid. The expression of the podocyte markers (*NPHS1, NPHS2, PODXL*) as well as the marker for proximal tubular cells (HNF4A) was significantly reduced upon the LPS treatment but was abrogated by MP. However, the expression level of the distal tubular cell marker *GATA3* and *CDH1* was not affected by the LPS or MP treatment (Figure 6A). This indicated that LPS-induced apoptosis might mainly occur in podocytes and proximal tubules.

We further co-localized the expression of cleaved Caspase-3, a marker of apoptosis, with markers of different cell types using immunofluorescence. The results showed that cleaved Caspase-3 was expressed mainly with SYNPO, a podocyte marker, and LTL, a proximal tubular marker (Figures 6B,C). While only a minority of cleaved Caspase-3 was colocalized with ECAD, a distal tubular marker (Figure 6D). The MP treatment partially rescued the LPS-induced apoptotic effects again. These findings suggested that LPS-induced apoptosis occurred primarily in podocytes and proximal tubular cells.

## Discussion

Sepsis-associated acute kidney injury is a life-threatening syndrome with high mortality (4). The two main key elements of SA-AKI include, as the name suggests, sepsis and AKI. Sepsis emphasizes infection and organ dysfunction, while AKI emphasizes acute kidney failure. Sepsis and AKI exert in turn, further susceptibility to each other, forming a vicious loop. The interaction between sepsis and AKI is complex. Hypotension has long been considered the main trigger for SA-AKI. However, recent findings indicated that multiple mechanisms might be involved in the development of SA-AKI, especially pathological changes in kidney parenchymal cells (7). The innate complexity of kidney constitution, as well as the enigmatic pathological mechanisms, urge the development of a new strategy to study the underlying pathogenesis, as well as to identify potential therapeutic targets for SA-AKI. In the present study, we successfully induced the kidney organoid consisting of podocytes and tubular cells of different segments. We applied LPS to induce the SA-AKI model in both *in vivo* and *in vitro* models. Our data suggested that LPS could directly induce oxidative stress and apoptosis in the kidney organoid, independent of changes in kidney perfusion and infiltration of immune cells.

Kidney organoids provide an improved platform for studying the mechanisms involved in kidney injury, compared with traditional *in vitro* cell culture models. Previous studies have shown the feasibility of using kidney organoids to study the mechanism of cisplatin-induced kidney injury (28). Kidney organoids allow for the detailed investigation of kidney parenchymal cells in the absence of other confounders such as changes in blood perfusion and immune cells. Meanwhile, kidney organoids, consisting of different cell types including tubular cells of different segments as well as podocytes, are superior to commercial cell lines, since they provide the possibility of revealing the intercellular crosstalk between kidney parenchymal cells. TLR4, the fundamental receptor of LPS, is found in a variety of innate immune cells and can promote various immune responses (29). In recent years, TLR4 has also been recognized as an important regulator of resident kidney cells (30). In the present study, TLR4 was found to be widely expressed in kidney organoids. The existence of various renal parenchymal cells, as well as TLR4, make kidney organoids a suitable platform for effectively studying LPS-related AKI.

Enhanced oxidative stress has been considered a predominant factor in the pathological process of AKI (31). Intensified oxidative stress is a crucial mediator of the innate immune response, which can later contribute to “cytokine storms” (32). Moreover, ROS overproduction can be an important trigger for parenchymal cell injury to the kidneys (25). Therefore, the state of oxidative stress may provide clues for the severity of SA-AKI. However, to our knowledge, one of the drawbacks of ROS evaluation in kidneys is the absence of suitable live *ex vivo* models. Moreover, it is difficult to distinguish whether ROS production is caused mainly by primary injury or by secondary immune cell influx in traditional animal models. In this study, in addition to MDA and SOD detection, we used a high-content imaging system to evaluate two oxidative stress parameters, DHE and Cellrox, in live kidney organoids. Both protein concentration and live imaging results indicated increased oxidative stress directly induced by LPS, in the absence of immune influx. However, the direct relationship between ROS and LPS-caused apoptosis has not been fully elucidated in our study, which may need further investigation.

Methylprednisolone, a member of the glucocorticoid family, is used as adjuvant therapy in sepsis treatment. In the current study, we found that MP treatment successfully reduced oxidative stress as well as the apoptotic process both *in vivo* and *in vitro*. In the kidney organoid experiments, we found that MP treatment could protect podocytes and tubular cells in the absence of immune cell influx. This indicated that the renoprotective role of MP in SA-AKI, at least partially, acted by influencing kidney parenchymal cells. It is well-known that glucocorticoids could exert different functions, either through the glucocorticoid receptor-mediated genomic actions or non-genomic actions. The different effects of glucocorticoid treatment are highly dependent on the dosage used. According to previous studies, a high dose of glucocorticoid treatment *in vitro* could induce cell apoptosis and oxidative stress (33–36), while a low dose could bring benefits (37, 38). In the present study, we confirm that treatment of the kidney organoid with 1 μg/mL of MP decreased the rate of apoptotic and oxidative stress in kidney cells. In recent years, the protective role of Nrf2 in kidney injury has been confirmed, due to its regulative effect on oxidative stress (39). In addition to antioxidant effects, Nrf2 has also been reported to be a positive regulator of some other physiological processes, such as DNA repair and mitochondrial function (27). In this study, the immunofluorescence staining of Nrf2 in kidney organoids confirmed the antioxidant effect of MP, while it revealed the possible mechanism by which MP performed its renoprotective role. However, there are still limitations, and we did not further investigate the therapeutic effects of treatment with different concentrations of MP. Furthermore, the detailed molecular mechanism of the protective role of MP in kidney parenchymal cells still needs further exploration.

In conclusion, we constructed an LPS-induced kidney injury model to simulate the development of SA-AKI. We observed increased immune cell infiltration and apoptosis of kidney cells in this model, which was reversed by MP treatment. We further applied kidney organoids derived from hPSC to study the toxicity of LPS in kidney cells. The LPS treatment led to increased oxidative stress and apoptosis in kidney organoids, and the MP treatment alleviated this effect. Therefore, we confirmed the efficacy of using kidney organoids as a new platform to study kidney injury and elucidated that LPS could exert a direct apoptotic effect on kidney parenchymal cells independently of inflammatory cell influx.

## Data Availability Statement

The original contributions presented in the study are included in the article/supplementary material, further inquiries can be directed to the corresponding authors.

## Ethics Statement

The animal study was reviewed and approved by the Animal Ethical Committee of Zhongshan Hospital, Fudan University (No. 2018-046).

## Author Contributions

WZ, MX, and TZ conceived and designed the study. WZ and RQ drafted and revised the manuscript. WZ and XZ performed the animal models. WZ performed kidney organoid differentiation. WZ, RQ, TL, XZ, and YS participated in the data acquisition, analysis, and interpretation. All authors approved the final version of the manuscript.

## Funding

This study was supported by the Shanghai Hospital Development Center (SHDC12018101 to MX and TZ) and the Zhongshan Hospital (Research Fund 203 to TZ).

## Conflict of Interest

The authors declare that the research was conducted in the absence of any commercial or financial relationships that could be construed as a potential conflict of interest.

## Publisher's Note

All claims expressed in this article are solely those of the authors and do not necessarily represent those of their affiliated organizations, or those of the publisher, the editors and the reviewers. Any product that may be evaluated in this article, or claim that may be made by its manufacturer, is not guaranteed or endorsed by the publisher.
